# Progress on Two-Dimensional Transitional Metal Dichalcogenides Alloy Materials: Growth, Characterisation, and Optoelectronic Applications

**DOI:** 10.3390/nano13212843

**Published:** 2023-10-27

**Authors:** Jia Yu, Shiru Wu, Xun Zhao, Zhipu Li, Xiaowei Yang, Qian Shen, Min Lu, Xiaoji Xie, Da Zhan, Jiaxu Yan

**Affiliations:** 1 Key Laboratory of Flexible Electronics (KLOFE), Institute of Advanced Materials (IAM), Nanjing Tech University, Nanjing 211816, China; 2 Changchun Institute of Optics, Fine Mechanics & Physics (CIOMP), Chinese Academy of Sciences, Changchun 130033, China; 3 University of Chinese Academy of Sciences, Chinese Academy of Sciences, Beijing 100049, China

**Keywords:** two-dimensional materials, transitional metal dichalcogenides, alloy phase

## Abstract

Two-dimensional (2D) transitional metal dichalcogenides (TMDs) have garnered remarkable attention in electronics, optoelectronics, and hydrogen precipitation catalysis due to their exceptional physicochemical properties. Their utilisation in optoelectronic devices is especially notable for overcoming graphene’s zero-band gap limitation. Moreover, TMDs offer advantages such as direct band gap transitions, high carrier mobility, and efficient switching ratios. Achieving precise adjustments to the electronic properties and band gap of 2D semiconductor materials is crucial for enhancing their capabilities. Researchers have explored the creation of 2D alloy phases through heteroatom doping, a strategy employed to fine-tune the band structure of these materials. Current research on 2D alloy materials encompasses diverse aspects like synthesis methods, catalytic reactions, energy band modulation, high-voltage phase transitions, and potential applications in electronics and optoelectronics. This paper comprehensively analyses 2D TMD alloy materials, covering their growth, preparation, optoelectronic properties, and various applications including hydrogen evolution reaction catalysis, field-effect transistors, lithium-sulphur battery catalysts, and lasers. The growth process and characterisation techniques are introduced, followed by a summary of the optoelectronic properties of these materials.

## 1. Introduction

The miniaturisation of silicon-based transistors is approaching its physical limits, posing challenges for integrated circuits in terms of fundamental physical principles and high-power consumption. To sustain Moore’s law, two-dimensional (2D) materials represented by transition metal dichalcogenides (TMDs) are expected to improve their high-density storage capabilities to achieve ultra-fast transmission characteristics. Their utilisation in optoelectronic devices is especially notable for surpassing graphene’s limitation of lacking a band gap. Moreover, TMDs offer advantages such as direct band gap transitions, high carrier mobility, and efficient switching ratios. The accurate tuning of electronic properties and band gaps in 2D semiconductor materials is vital for enhancing their overall performance.

**Figure 1:**
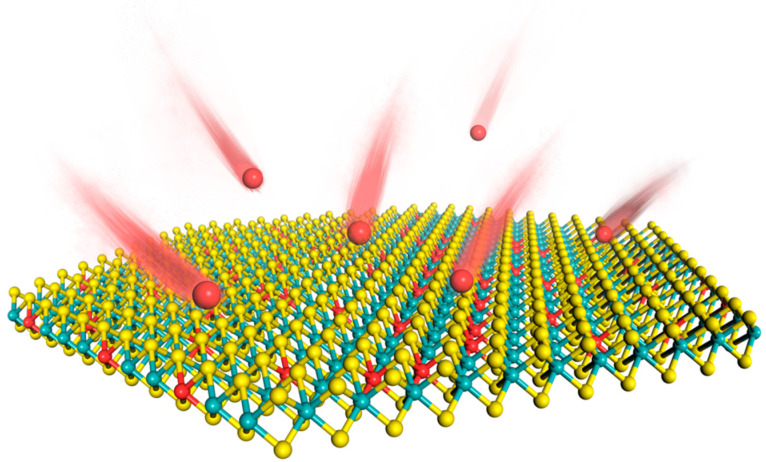
Creation of 2D alloy phases of TMD materials by doping with heteroatoms.

Researchers have explored the creation of 2D alloy phases through heteroatom doping, a strategy employed to fine-tune the band structure of these materials (Figure 1). The precise tuning of energy bands achieved by alloying engineering has led to the emergence of some photoelectric properties of 2D TMDs alloy materials. These attributes either remain absent in the original TMD materials or exhibit an elevated degree of excellence [1,2,3,4,5]. Their vibrational modes and photoluminescence peaks are affected by the alloying engineering, and this attribute provides a new direction for the application of 2D TMDs alloy materials in hydrogen evolution reaction (HER) [6,7,8], field-effect transistors (FET) [9,10], lithium-sulphur battery catalysis [11,12], and lasers [13,14]. As the exploration of alloyed 2D TMD materials advances, its role in substantiating prospective strides in multifunctional materials for ensuing generation technologies becomes a discernible projection.

In this review, we first introduce the preparation and characterisation methods of 2D TMDs alloy materials. Then, we focus on their development and potential in various application areas. We look into the future of 2D TMDs alloy materials and point out the bottlenecks in their development.

## 2. Preparation and Characterisation of 2D TMDs Alloy Materials

The following sections present a methodical outline of the techniques used for the preparation and characterisation of 2D alloy materials. The study begins by presenting various preparation processes, including mechanical stripping, chemical vapour deposition (CVD), physical vapour deposition (PVD), and chemical vapour transport (CVT). The advantages and disadvantages of each method are then compared and analysed. In addition, this article presents an introduction to the characterisation methods for 2D alloy materials. These methods include techniques for morphological analysis characterisation and spectral properties characterisation.

### 2.1. Preparation of 2D TMDs Alloy Materials

In laboratory settings, the preparation of 2D materials can be categorised into two primary methods: (1) Top-down, i.e., slowly tearing the lumpy layered TMDs material into a single- or few-layer material through mechanical peeling (dry method) and liquid phase etching substrate peeling (wet method). In the context of 2D semiconductor alloy materials, the typical approach involves synthesising the desired crystalline material through the CVT method. Subsequently, the material is subjected to shearing processes to yield single- or few-layer 2D materials. The disadvantage is that, in general, it is time-consuming and inefficient, and the final 2D layered material has a small area, which makes it not easy to control the uniform thickness of the sample, and the reproducibility is poor, making it difficult to operate in batch. (2) Bottom-up, including CVD and molecular methods. This method makes up for the shortcomings of the “top-down” approach and allows the preparation of large areas, high crystallinity, and a continuous atomic layer thickness of 2D TMDs. The CVD technique is presently the primary method employed in laboratories for producing 2D alloyed few-layer materials. This is due to its ability to yield high crystallinity, good homogeneity, and high controllability of samples [15]. Nevertheless, the method’s non-universal applicability is attributed to its limitations in meeting certain growth requirements.

#### 2.1.1. Mechanical Stripping Method

**Figure 2:**
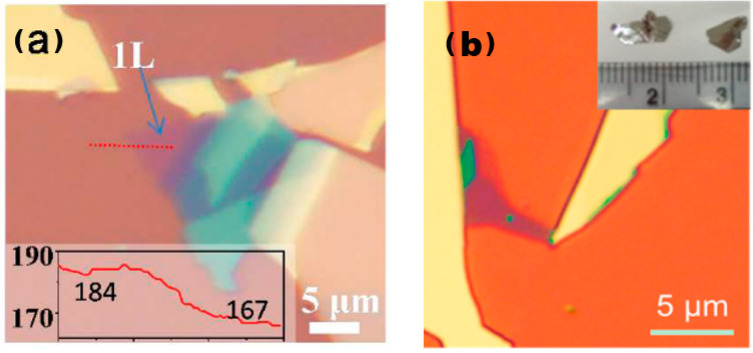
Atomic force microscopy images of 2D TMDs alloys prepared through mechanical exfoliation. (**a**) Optical image of exfoliated Mo_0.47_W_0.53_S_2_ flakes on Si/SiO_2_ substrate. Monolayer region is labelled as “1L”. The inset shows red colour intensity along the red dashed line, giving a contrast of (184–167)/184 = 9.2% for the 1L. Reproduced with permission [16]. Copyright 2013, American Chemical Society; (**b**) optical image of Mo_0.67_W_0.33_Se_2_ sheets. Inset in panel (**b**) is a photograph of Mo_0.67_W_0.33_Se_2_ single crystals. Reproduced with permission [17]. Copyright 2014, American Chemical Society.

The process of mechanical peeling involves the utilisation of an adhesive material, such as tape, to bind together crystalline materials. By applying mechanical force, the weak van der Waals forces between the layers of crystalline materials are disrupted, resulting in the thinning of the material. This results in the desired final single- or few-layer sample, as illustrated in Figure 2 [16,17].

The mechanical peeling method is not significantly influenced by the material properties, and therefore, any block TMDs material that is prepared using the universal CVT method can be utilised to obtain the desired sample through this technique. Furthermore, since the prepared crystalline material is very crystalline and the peeling of the sample only destroys the van der Waals interactions between the layers, the surface of the final few-layer sample is not affected by problems such as improper handling during the preparation process, let alone the contamination of its surface by solvents, due to the fact that the whole process of mechanical peeling is a dry process. Therefore, mechanical peeling is often used in the study of the intrinsic properties of materials, making it one of the most important preparative tools in the study of the properties and applications of 2D materials.

However, in the process of sample preparation using the mechanical peeling method, the peeling by tape is very rough, and we can understand that the size, area, and thickness of the material cannot be perfectly adjusted precisely, especially in materials with strong interlayer van der Waals forces. It becomes more difficult to adjust the thickness by using this method. In addition, the size of the few-layer samples prepared through mechanical peeling is very small, which cannot fully satisfy the daily experimental conditions, and the thickness and size distribution of the samples on the substrate is also random, which is not conducive to large-scale application in electronic devices. In addition, the mechanical peeling of the sample and the substrate also becomes part of the residual glue, which also produces a certain degree of contamination. Although it can be erased through thermal annealing in a vacuum, this operation inevitably causes damage to the sample. In summary, these undesirable factors limit the prospects of the mechanical peeling method for the preparation of few-layer samples.

#### 2.1.2. Chemical Vapour Deposition

**Figure 3:**
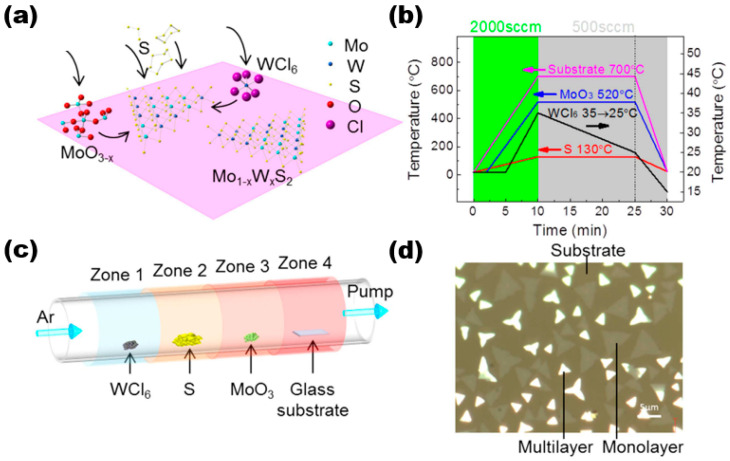
LP-CVD synthesis of monolayer Mo_1−x_W_x_S_2_. (**a**) Schematic illustration of the surface reactions during CVD growth; (**b**) the arrangement of WCl_6_, sulphur, MoO_3_, and substrate in the CVD system. They were placed from upstream to downstream in four different furnace zones. The temperatures of each zone could be controlled independently. (**c**) Temperature profiles of the precursors and the substrate during CVD growth. The temperatures of each precursor and the substrate rose to their designated values within 10 min under 2000 sccm Ar flow rate. Then, the Ar flow was switched to 500 sccm for film growth. The temperature of the substrate, MoO_3_, and S sources were kept constant while the temperature of WCl_6_ gradually dropped from 35 to 25 °C in 15 min before cooling down. (**d**) Optical image showing that the triangular flakes grew on the glass substrate (dark regions). Brighter ones are the multilayers, and less bright ones are monolayers. Reproduced under the terms of the CC-BY 4.0 license [18]. Copyright 2016, The Authors, published by Springer Nature.

The CVD method is a widely used technique for producing thin film materials. This method involves the chemical reaction of one or more gas-phase molecules on the surface of a substrate, resulting in the formation of thin film materials. It is generally divided into three reaction processes, namely, the formation of gas-phase molecules, the transfer of gas-phase molecules to the deposition area, and a reaction to form the target thin film sample. Here, we discuss and study the preparation of alloy materials by using the CVD method using Mo_1−x_W_x_S_2_ as an example. In their study, Wang et al. utilised the low-pressure chemical vapour deposition technique to synthesise high-quality thin film materials of Mo_1−x_W_x_S_2_ [18]. Figure 3a shows the reaction schematic of the sample prepared by using the CVD method. They used three precursors and substrates placed in the reaction system in a certain order, with components, purity, and content as follows: WCl_6_ (99.9%, ~5 mg), sulphur (99%, 1.0 g), MoO_3_ (>99.5%), and substrate (slide S1214). They were placed in a quartz tube with a diameter of 2 inches in the order shown in Figure 3c. The experiment utilised high-purity argon gas as the carrier gas. Four distinct temperature zones were employed to regulate the temperature of various precursors. The argon flow rate was adjusted to control the pressure. The temperature profile plot in Figure 3b illustrates the temperature distribution. There were three main stages. The first was the first 10 min of the warming stage, where the four precursors were warmed up to the set value temperature, i.e., WCl_6_ was 35 °C, sulphur was 130 °C, MoO_3_ was 520 °C, and the substrate was 700 °C, corresponding to an argon flow rate of 2000 sccm. Then, the next 15 min was the growth stage of the target film sample; here, it should be noted that WCl_6_ grew gradually. During the cooling phase of the CVD reaction, WCl_6_ was rapidly cooled to 15 °C for the final 5 min. Subsequently, the remaining components were cooled to room temperature once the entire CVD reaction had concluded. The final samples were processed, and they were transferred using a standard methyl methacrylate (PMMA)-assisted transfer method by spin-coating with PMMA, effectively separating the samples on the glass substrate with aqueous NaOH solution, then salvaging the PMMA-coated Mo_1−x_W_x_S_2_ film samples through other substrates, and dissolving the PMMA using acetone. The thin film sample material was acquired as the final target (as shown in Figure 3d). The multilayer sample is represented by the bright area, while the monolayer sample is indicated by black lines in the figure. The substrate is depicted by the light brown area.

It should be noted that in this project, although the growth temperature of WS_2_ was reduced to very close to that of MoS_2_ by using highly volatile WCl_6_ as a precursor, we could not completely avoid the formation of MoS_2_/WS_2_ heterostructures. This highlights a challenge in producing superior alloy materials using the CVD technique. In the process of growing 2D materials, the challenge of identifying source materials that can volatilise at lower temperatures results in a significant proportion of materials that cannot be synthesised through the CVD technique. Liu et al. [19] proposed that the molten salt-assisted method can be utilised to obtain the majority of TMDs 2D materials. The process reduces the melting point of metal precursors and facilitates their reaction with oxides to produce metal-aluminium oxides that are volatile. This enhances the accessibility of metal precursors to the gas phase, which simplifies the reaction. While the CVD method shows promise for producing metal sulphides, the resulting samples are currently inferior to those produced by using the stripping method. On the other hand, compared with the CVT method, CVD is not universal and is slightly inadequate in studying the intrinsic properties of materials.

#### 2.1.3. Physical Vapour Deposition

**Figure 4:**
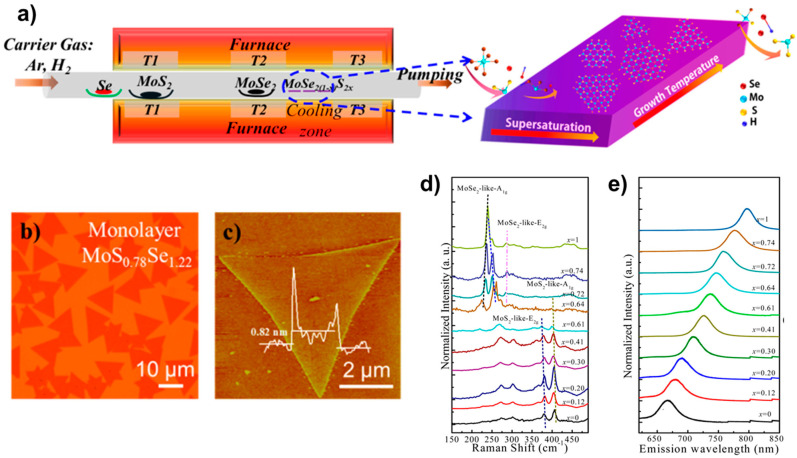
(**a**) Illustration of monolayer MoS_2(1−x)_Se_2x_ growth. (**b**) Optical image, (**c**) AFM image, (**d**) the composition-dependent Raman spectra, and (**e**) the composition-dependent photoluminescence (PL) spectra. Reproduced with permission [20]. Copyright 2015, American Chemical Society.

The PVD is one of the easiest means to prepare alloy materials. In 2015, MoS_2(1−x)_Se_2x_ alloy materials were prepared by using the PVD method by Feng et al. [20]. In contrast to the growth conditions previously designed [21], the researchers were able to effectively suppress the decomposition of MoSe_2_ under high-temperature conditions by enhancing the experimental conditions. This was achieved by introducing Se, which was enriched during the reaction. The entire reaction process is illustrated in Figure 4a. The process was carried out by placing the selenium powder in the upstream zone of the tube furnace with the temperature set at 300 °C; then, the powders of MoS_2_ and MoSe_2_ were placed in the order shown in the figure and evaporated by a high temperature (about 950~965 °C). The SiO_2_/Si substrate was placed in the precipitation zone with a lower temperature (about 600~770 °C) for the growth of the target sample material. Finally, large-size highly crystalline MoS_2(1−x)_Se_2x_ (x = 0.41~1.00) monolayer alloy samples of different components were achieved. Figure 4b shows the optical images of the prepared MoS_0.78_Se_1.22_ monolayer sample, whose atomic force microscopy (AFM) images were fingerprinted on the monolayer sample. Furthermore, an analysis was conducted on the Raman spectra and fluorescence spectra of the material, which can be observed in Figure 4e,f. Due to the interrelation between the fluorescence spectra and band gap engineering properties of 2D TMD materials, it is imperative to emphasise the significance of fluorescence spectra in this context. The team verified the link between the forbidden band width and the degree of alloying of the alloyed material by using fluorescence spectroscopy, and they succeeded in achieving an effective modulation of the band gap from 1.86 to 1.55 eV (Figure 4f). A comparable technique was employed to effectively synthesise Mo_1−x_W_x_Se_2_ [17]. This resulted in a modulation of the band gap width from 1.56 to 1.65 eV.

The PVD method plays a crucial role in investigating the characteristics and uses of TMDs materials. However, the method’s effectiveness is heavily dependent on the source material’s properties, and there are limited controllable parameters throughout the synthesis process. Consequently, several materials cannot be synthesised successfully using this method, leading to a lower level of universality when compared to the CVD method. In contrast, the quality of samples produced through PVD is comparatively inferior to that of materials fabricated using the CVD technique. The constraints associated with PVD make it challenging to achieve widespread adoption in the realm of TMDs.

#### 2.1.4. Molecular Beam Epitaxy (MBE)

The molecular beam is epitaxially grown on the substrate in the form of a scan, so it can be seen as a slow “stacking” of molecules into a crystalline material. In this method, the sample is grown layer by layer, so the growth rate is very slow, and the number of layers of material can then be controlled very carefully and precisely. Zhang et al. [22] conducted a related study using 2D V_x_Mo_1−x_Se_2_ prepared through molecular beam epitaxy. The MBE technique requires the material to be grown on a conductive substrate, which poses a limitation for in situ characterisation of the resulting samples. This is a well-known constraint of the MBE process. On the other hand, the growth conditions for sample preparation using the MBE method are very stringent and expensive, and the preparation on a large scale is influenced by the size of the cavity and substrate, so the MBE method is unsuitable for synthesising materials over large areas. Furthermore, the crystals produced through MBE are relatively diminutive, impeding the investigation of superior materials for practical purposes.

#### 2.1.5. Atomic Layer Deposition (ALD)

**Figure 5:**
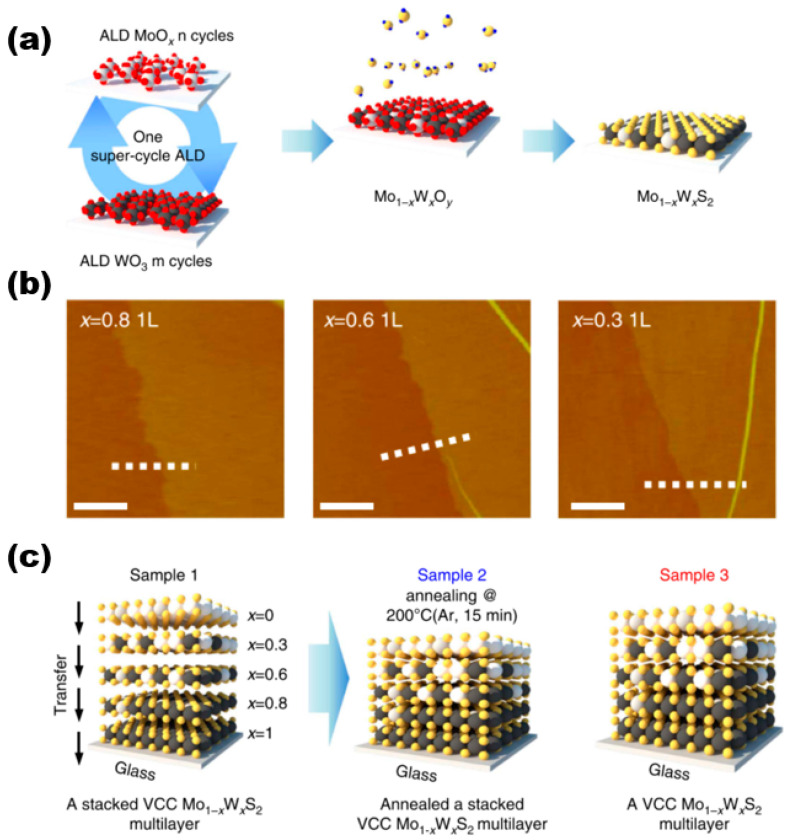
(**a**) Synthesis procedure of super-cycle ALD for Mo_1−x_W_x_S_2_ alloy. (**b**) AFM images of composition-dependent molybdenum disulphide tungsten alloy materials. (**c**) Schematics of three sample types for ultraviolet–visible spectrophotometer measurement. Reproduced under the terms of the CC-BY 4.0 license [23]. Copyright 2015, The Authors, published by Springer Nature.

In a paper published by Song et al. [23] in 2015 on the preparation of molybdenum disulphide tungsten alloy materials for controlled growth, they used ALD for the synthesis of the materials, and the schematic diagram of their overall preparation is shown in Figure 5a. In this work, they first performed a hypercyclic atomic layer deposition preparation process constructed by n ALD cycles of MoO_x_ and m ALD cycles of WO_3_, and the deposited Mo_1−x_W_x_O_y_ alloy film samples were sulfidised. Different cycles were used for MoO_x_(n) and WO_3_(m) in one overall cycle to deposit 0.8–0.9 nm thick composition-controlled Mo_1−x_W_x_O_y_ alloy films, which in turn led to the preparation of monolayer Mo_1−x_W_x_S_2_ alloy samples (AFM images are shown in Figure 5b). They all exhibited good homogeneity and continuity. The thicknesses of Mo_0.2_W_0.8_S_2_, Mo_0.4_W_0.6_S_2_, and Mo_0.7_W_0.3_S_2_ were all 0.1 nm, which corresponds to the monolayer Mo_1−x_W_x_S_2_ alloys. Bilayer and trilayer Mo_1−x_W_x_S_2_ samples were also successfully prepared by using this method. The study conducted an evaluation of the interlayer coupling effects on the interlayer transition process [24,25,26,27] in the synthesised multilayers of vertically composition-controlled Mo_1−x_W_x_S_2_ materials. This evaluation was based on a comparison of the interlayer transitions of three different sample types, as illustrated in Figure 5c. Although these sample types are not particularly relevant to the paper, the study found that strong interlayer coupling effects were present.

ALD is a method that produces high-quality crystals. However, the equipment required for this process is expensive and not universally applicable. Additionally, the deposition rate of the ALD method is slow, which limits its use and commercial industrial applications.

#### 2.1.6. Chemical Vapour Transport

**Figure 6:**
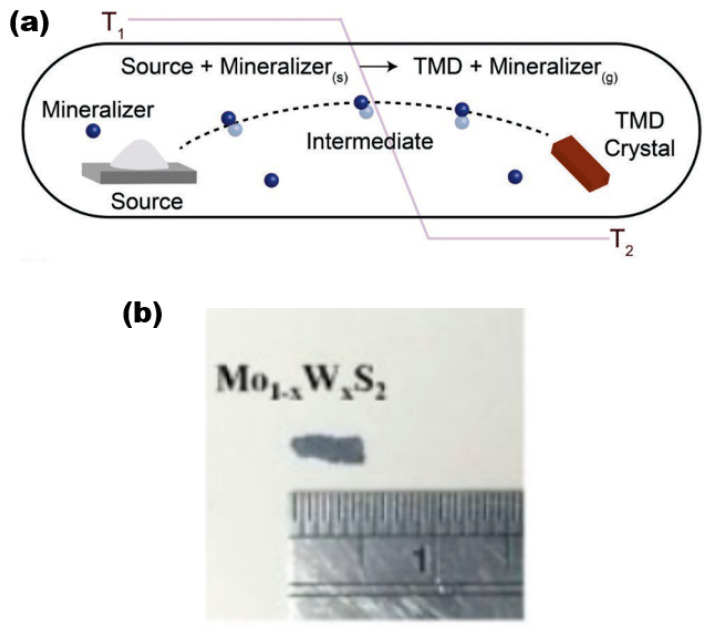
(**a**) Schematic diagram of the reaction process of the CVT method. Reproduced with permission [28]. Copyright 2019, Wiley-VCH. (**b**) Mo_1−x_W_x_S_2_ sample pictures. Reproduced with permission [30]. Copyright 2017, IOP Publishing on behalf of the Japan Society of Applied Physics (JSAP).

The CVT is a widely used synthesis technique in the laboratory for producing 2D alloy materials. CVT reactions involve a solid material that is not readily vapourised and can react with a gas-phase reactant to form a gaseous product. The gaseous product then deposits the crystalline material in the material growth zone. This gas-phase reactant is called a transport agent (Figure 6a) [28], which shows a typical synthesis of TMD crystals. To ensure successful reaction progression, it is imperative to regulate the temperature within both the reaction and growth zones. This necessitates the creation of a specific temperature gradient, which is crucial for the proper execution of the experiment. In the reaction zone, the solid (powder) and the transport agent react in the gas phase. then slowly diffuse into the growth zone, and deposit in its region, gradually forming the crystalline material (Figure 6b,c) [29]. It is worth noting that the formation process of bulk TMD crystalline materials is, as a rule, a heat absorption process, so the temperature T_1_ (sublimation temperature) is set higher than T_2_ (deposition temperature) in most cases. The CVT method is widely recognised as a dependable and effective means of producing bulk TMD crystalline materials for both scientific and commercial purposes.

The following is a discussion of the preparation process for Mo_1−x_W_x_S_2_ single crystals using the CVT method [30] as an example. To prepare Mo_1−x_W_x_S_2_ crystalline material, the solid reactants generally used are molybdenum powder (99.99% purity), tungsten powder (99.99%), and sulphur powder (99.999%), with iodine gas as a transport agent. The quartz tubes used were 20 cm × 22 mm × 17 mm in size (corresponding to tube length, outer diameter, and inner diameter, respectively), cooled in liquid nitrogen, evacuated to 10^−6^ Torr, and sealed. The tubes underwent pre-reaction at a temperature of 1000 °C for a duration of 2 weeks. Subsequently, they were subjected to a three-zone furnace with a temperature gradient ranging from 960 to 1050 °C. This was carried out to ensure that the growth conditions of the desired samples were met. Finally, after a growth cycle of about one month time, the furnace was slowly cooled down to room temperature. Finally, the large-size target sample was obtained in the growth zone (Figure 6b), and the size of the prepared crystalline Mo_1−x_W_x_S_2_ sample was about 2 × 5 mm^2^ with a thickness range of 3–6 μm.

The selection of the transport agent is a crucial factor in the advancement of the entire CVT method reaction process. Iodine gas, hydrogen halide, and hydrogen are commonly selected as transport carriers for most TMDs. The basis for their selection is closely related to the inherent stability of the solid reactants [31].

Mechanical stripping, CVD, PVD, MBE, ALD, and CVT mentioned above are all commonly used methods for the preparation of 2D TMDs alloy materials. It is worth noting that the specific details of the preparation process may vary significantly depending on the selected TMD alloy, synthesis method, and research objectives. The properties of the resulting materials are optimised by exploring different growth conditions and techniques for specific applications such as catalysis, electronics, or photonics.

### 2.2. Characterisation Methods for 2D TMDs Alloy Materials

The laboratory characterisation process for 2D alloy materials involves methods such as optical microscopy (OM), scanning electron microscopy (SEM), atomic force microscopy (AFM), X-ray diffraction (XRD), X-ray photoelectron spectroscopy (XPS), Raman spectroscopy, photoluminescence (PL) spectroscopy, and other similar techniques. These methods are used to determine the size, shape, thickness, and composition of the prepared 2D alloy materials, similar to traditional 2D material characterisation methods.

#### 2.2.1. Morphological Structure Analysis

1. Optical microscope

**Figure 7:**
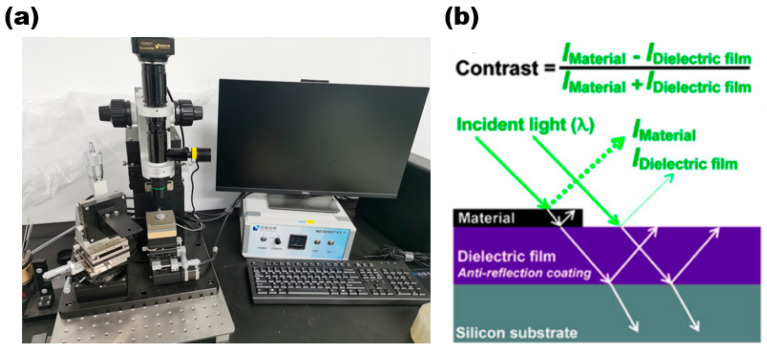
(**a**) Two-dimensional Transfer System with Optical Microscope; (**b**) optical schematic diagram of optical microscope. Reproduced with permission [32]. Copyright 2017, American Chemical Society.

The optical microscope enables the initial observation of the sample’s distribution on the substrate and facilitates the easy and quick acquisition of information on the sample’s shape and size. Figure 7a is a 2D material transfer system (with a metallographic optical microscope) of Nanjing Metatest corporation. The optical schematic diagram of the optical microscope is shown in Figure 7b.

**Figure 8:**
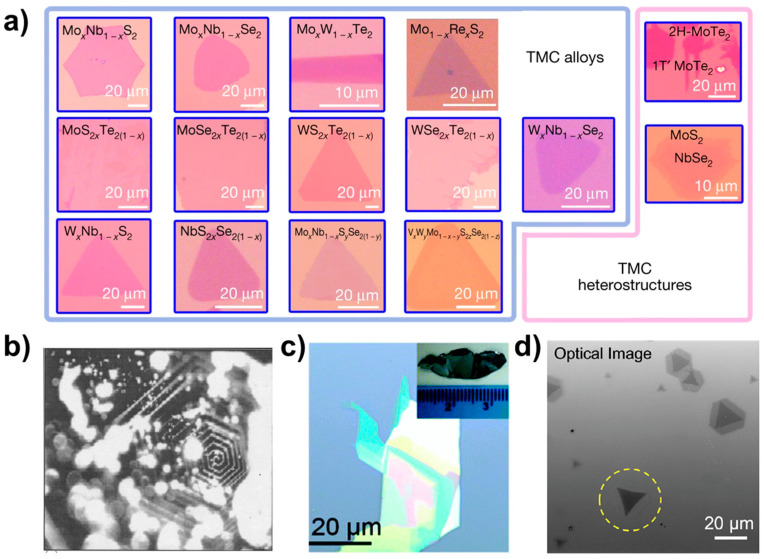
Optical images. (**a**) Optical photos of TMCs alloy materials and heterostructures. Reproduced with permission [19]. Copyright 2018, Springer Nature. (**b**) MoS_2_. Reproduced with permission [33]. Copyright 2004, Indian Academy of Sciences. (**c**) SnSe_0.5_S_1.5_. Reproduced with permission [34]. Copyright 2017, Royal Society of Chemistry. (**d**) Optical image of MoS_2_-WS_2_ lateral heterostructures. The laser-irradiated flake is marked with a yellow dashed circle. Reproduced with permission [35]. Copyright 2021, American Chemical Society.

Optical microscopy plays a crucial role in the initial examination of a sample microstructure and serves as a tool for characterising the underlying morphology. By utilising OM, we can obtain fundamental information about the sample surface, such as size and morphology. Figure 8 shows 2D optical photographs of materials taken by researchers using optical microscopy [19,33,34,35]. In Figure 8a are the ternary alloy materials MoS_x_Te_2−x_, MoSe_x_Te_2−x_, WS_x_Te_2−x_, WSe_x_Te_2−x_, NbS_x_Se_2−x_, Mo_x_N_1−x_S_2_, Mo_x_Nb_1−x_Se_2_, Mo_1−x_Re_x_S_2_, W_x_Nb_1−x_S_2_, W_x_Nb_1−x_Se_2_, and Mo_x_W_1−x_Te_2_, the quaternary alloy Mo_x_Nb_1−x_S_2y_Se_2(1−y)_, the quintuple alloy material V_x_W_y_M_1−x−y_S_2z_Se_2(1−z)_, and the optical pictures of the 1T’-MoTe_2_/2H-MoTe_2_ and MoS_2_-NbSe_2_ heterostructures. The optical photographs presented herein depict the surface morphology of the bulk crystalline material. Figure 8b displays a photograph of a single crystal of MoS_2_, showcasing a hexagonal spiral shape that is associated with the material growth mechanism. By examining the sample photographs captured via an optical microscope, one can obtain preliminary information regarding the sample’s shape, size, and other characteristics. In addition, from the obvious optical contrast lining for few-layer materials, such as in the optical photograph of SnSe_0.5_S_1.5_ nanosheets in Figure 8c and the photograph of a single crystal of SnSe_0.5_S_1.5_ shown in the inset, we can predict the thickness of the sample, i.e., the darker areas have a large thickness under the optical microscope, while the relatively brighter areas have a smaller thickness.

Figure 8d illustrates the heterogeneous structure of MoS_2_-WS_2_. The monolayer depicted in the figure displays a distinct optical contrast in the area highlighted by yellow circles as compared to other regions.

2. Atomic force microscopy

**Figure 9:**
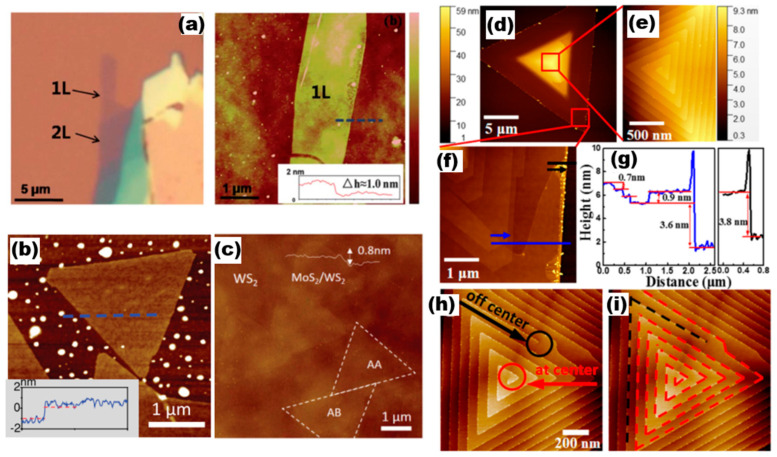
AFM images with the line profiles. (**a**) Optical micrograph and AFM of Mo_0.47_W_0.53_S_2_ flake. Reproduced with permission [36]. Copyright 2014, Royal Society of Chemistry. (**b**) AFM image of MoS_1.60_Se_0.40_. Reproduced with permission [21]. Copyright 2014, Wiley-VCH. (**c**) AFM image of MoS_2_/WS_2_. Reproduced with permission [38]. Copyright 2016, Wiley-VCH. (**d**–**i**) AFM image of WS_2_ a single spiral structure. Reproduced with permission [37]. Copyright 2018, American Chemical Society.

AFM has the capability to not only investigate the surface morphology of a sample but also to assess its mechanical characteristics, including hardness, elasticity, and plasticity. AFM has the characteristics of high accuracy, low damage to the sample, and wide range of use, making it widely used. In general, the working modes of AFM testing include contact, non-contact sub, and click type. This subsection presents AFM images of various 2D TMDs materials that are commonly used. The images are illustrated in Figure 9. The optical pictures and AFM images of the Mo_1−x_W_x_S_2_ monolayer alloy studied by Chen et al. [36] are shown in Figure 9a, where the layers of the alloy film sample were identified using AFM measurements. This included one layer, two layers, and the thickness of the monolayer alloy sample of approximately 1.0 nm (inset in a). Figure 9b,c show AFM images of MoS_1.60_Se_0.40_ monolayer semiconductor alloys [37] and AFM images of MoS_2_/WS_2_ heterostructures with a thickness of about 0.8 nm, respectively [38]. The formation mechanism of helical WS_2_ [37] was probed by Fan et al., and the AFM images they constructed are presented in Figure 9d–i. The central region of Figure 9d reveals an obvious triangular helical morphology after the magnification of Figure 9e The AFM of the magnified Figure 9c, focusing on its edge dark region, indicates that the heights of these steps are approximately 0.7 nm, 0.9 nm, 3.6 nm, and 3.8 nm, and the growth process of the AFM image with such a representative helical dislocation, i.e., terminated at the central/eccentric position, was investigated based on the AFM images (Figure 9h,i marked with red lines).

AFM is capable of providing clearer and more intuitive sample information compared to optical microscopy. It boasts a highly sensitive surface-state-sensing capability, allowing for the probing of material surface information with exceptional clarity and rapid precision. It brings convenience to studying the thickness of 2D alloy materials. Meanwhile, compared with the STM characterisation method, AFM requires a smaller operating environment and is not affected by the sample conductivity. Based on these advantages, AFM has been widely used [39]. AFM has made significant advances in characterising Fermi levels and hidden interfaces in materials, with techniques such as scanning Kelvin probe force microscopy (SKPFM) [40,41,42], Kelvin probe force microscopy (KPFM) in ultrahigh vacuum [43,44], etc., which have enabled researchers to probe electronic properties and surface structures at the nanoscale.

3. Scanning Electron Microscopy

The scanning electron microscopy (SEM) offers the benefit of a wider scanning field of view and the capability to focus on samples ranging from the nanometre to the millimetre scale. The SEM provides more comprehensive sample information compared to optical microscopy. It can observe the geometry, dispersion state (powder), particle size, and distribution of the sample being measured. Additionally, it can determine the elemental composition or phase structure of a specific morphology in a specific region. This information is not available through optical microscopy [45]. In conclusion, the comprehensive nature of SEM presents a promising future for the investigation of the physical properties of materials.

**Figure 10:**
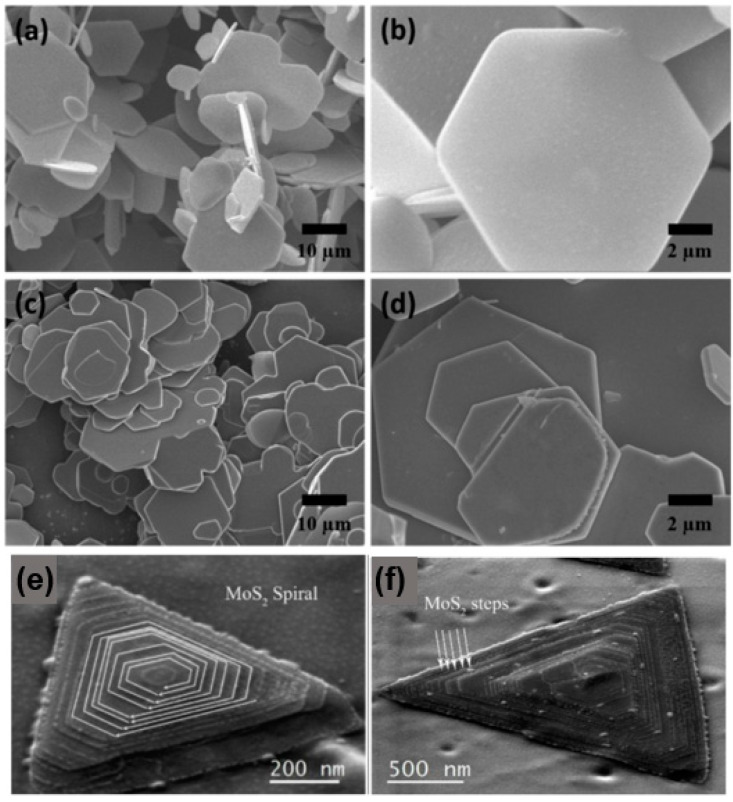
SEM images. (**a**) Low magnification and (**b**) high magnification SEM images of MoS_1.5_Se_0.5_. (**c**) Low magnification and (**d**) high magnification SEM images of WS_1.62_Se_0.38_; (**e**) SEM image of MoS_2_; and (**f**) SEM image of a MoS_2_ showing the multiple layered steps. (**a**–**d**) Reproduced with permission [45]. Copyright 2017, Elsevier. (**e**,**f**) Reproduced with permission [46]. Copyright 2017, Springer Nature.

The SEM images of the synthesised MoS_1.5_Se_0.5_ and WS_1.62_Se_0.38_ ternary alloy samples are presented in Figure 10. The images depict the samples at low and high magnification [45]. It can be clearly seen that both MoS_1.5_Se_0.5_ and WS_1.62_Se_0.38_ show layered features and have the same structure, i.e., they are composed of hexagonal nanoflakes with smooth surfaces. The pyramidal helical structure of MoS_2_ is fully visible in Figure 10e, with the clockwise growth direction indicated by the white arrow. The SEM in Figure 10f shows that we can observe the different “steps” in the formation of the “pyramid” configuration, which are marked in the figure, and the edge areas are defects of the sample, which lead to the deviation of the sample from the standard perfect triangle. The SEM is a useful tool for analysing samples, providing information on their structural characteristics, surface dimensions, and crystallinity. Additionally, the SEM can be used to study the distribution of elements in specific micro-regions, determine the percentage of each element present, and gather other relevant data. This information is particularly valuable for investigating the structure, growth mode, and properties of 2D materials. It is involved in the examination of the configuration, development pattern, and characteristics of 2D substances.

#### 2.2.2. Optical Characterisation

1. Raman spectroscopy

The Raman effect, also known as Raman scattering, was first observed by Chandrasekhara Venkata Raman, an Indian physicist, in 1928. In recognition of his contributions to the field of optics, he was awarded the Nobel Prize in Physics in 1930.

**Figure 11:**
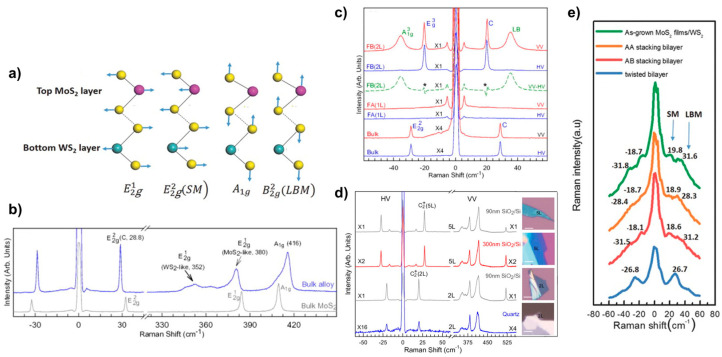
(**a**) Schematic diagram of lattice structure and vibrational mode for bilayer heterostructure WS_2_/MoS_2_. (**b**) Raman Spectra of Bulk MoWS_2_ and MoS_2_; (**c**) low-wavenumber Raman in Polarised Mode of bulk, 1L, and 2L alloy flake MoWS_2_; (**d**) polarisation Raman spectra of few-layer MoWS_2_ on different substrates; and (**e**) low-frequency Raman spectroscopy of layer breath mode and shear mode. (**a**,**e**) Reproduced with permission [38]. Copyright 2016, Wiley-VCH. (**b**–**d**) Reproduced with permission [48]. Copyright 2015, AIP Publishing.

Raman spectroscopy is a convenient and fast technique to study the properties of 2D TMDs materials because of its ease of operation, fast measurement, low maintenance cost, high resolution, lack of contact and damage to the sample, the ability to test at low temperatures and high pressures, accurate imaging, and the ability to provide information on the lattice structure and electronic structure of the measured sample. The Raman characteristic peaks of 2D materials are affected by their coupling properties, resulting in their appearance or shift. At wave numbers below 50 cm^−1^, the phonon shear mode (SM) and layer breath mode (LBM) can be directly observed. This technique has also been widely applied to study the stacking behaviour of 2D van der Waals heterojunctions [47]. Zhang et al. [38] have identified new phonon modes, SM and LBM, in a stacked configuration of bilayer heterostructures such as MoS_2_/WS_2_. These modes are observed in structures with strong interlayer coupling forces and correspond to relative and mutual vertical oscillations of the rigidity between adjacent layers of a bilayer heterogeneous structure. Figure 11a illustrates these modes, where SM and LBM represent the former and the latter, respectively. The interaction between these modes can be likened to a “spring” connecting two sulphur layers. These phonon modes are particularly important for investigating the thickness of the sample layers, the way the samples are stacked, the surface adsorption, and the contact quality of the 2D material hybridisation. Figure 11e presents the low-wavenumber Raman spectra of MoS_2_ films on WS_2_, AA stacking mode, AB stacking mode, and corner heterojunctions. It can be clearly seen that the Raman shift of the A-B stacked LBM is increased by 2 cm^−1^ compared to the A-A stacked bilayer structure, which can be attributed to the shorter interlayer distance and stronger interlayer coupling force of the A-B stacked approach. It can also be seen that the LBM vibration frequency of the corner heterojunction MoS_2_/WS_2_ is significantly lower by 2–4 cm^−1^ compared to that of the A-B stacked MoS_2_/WS_2_, which indicates weaker interlayer coupling and lower stacking efficiency.

Moreover, the interconnection of individual monolayers in 2D materials is facilitated by van der Waals interaction forces. The characteristics of multilayer alloys, on the other hand, are dependent on the number of layers they possess. Qiao et al. [48] conducted a study on the linkage between the number of layers of an alloy and its sheer mode and layer breathing mode. As shown in Figure 11b, the Raman peak at 416 cm^−1^ is designated as $A_{1g}$ due to the spatial point group type D6h of the bulk alloy, which is located between MoS_2_ (409 cm^−1^) and WS_2_ (420 cm^−1^) [49]. In contrast, the MoS_2_-like and WS_2_-like alloys, $E_{2g}^{1}$, are represented at 380 cm^−1^ and 352 cm^−1^ Raman shifts, respectively, and an ultralow-frequency (ULF) Raman peak is observed at 28.8 cm^−1^. The SM mode in the bulk alloy is responsible for this attribute. Figure 11c demonstrates the observation of the ULF peak under polarisation. The FWHM in the bulk alloy is approximately 1.1 cm^−1^, which is consistent with the bulk MoS_2_.

The above studies have shown that LBM and SM vibrations in low-wavelength-number Raman spectroscopy can be used as a fingerprint characterisation of stacking configurations of 2D materials. There is also a large body of work on Raman studies of Mo_1−x_W_x_S_2_ 2D alloy materials doped with X [36] and at different temperatures [50].

2. Photoluminescence spectra

**Figure 12:**
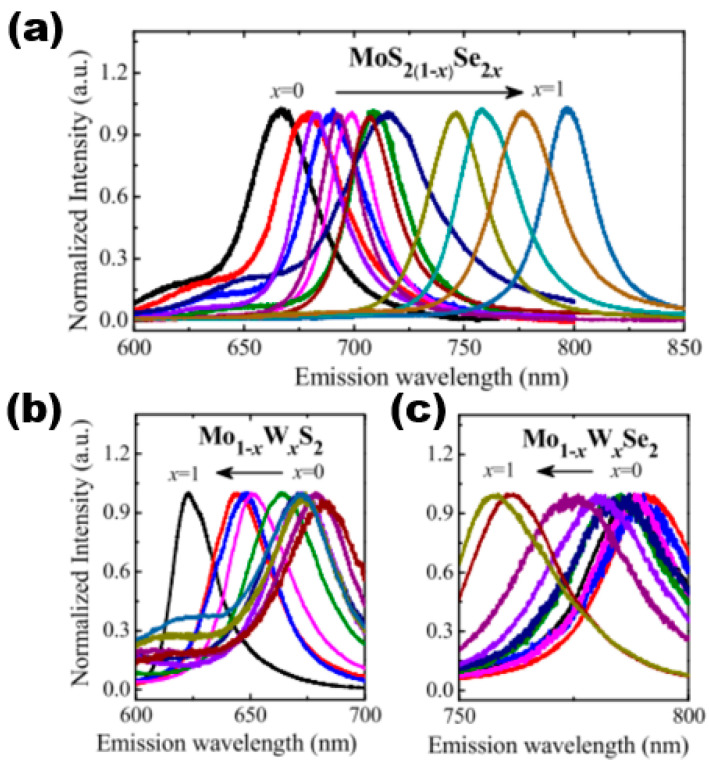
Composition-dependent PL spectra of (**a**) MoS_2(1−x)_Se_2x_ monolayers, (**b**) Mo_1−x_W_x_S_2_ monolayers, and (**c**) Mo_1−x_W_x_Se_2_ monolayers in the whole composition range (x = 0–1). Reproduced with permission [51]. Copyright 2015, The Royal Society of Chemistry.

When a high-density energy laser beam is directed onto the surface of a sample, it results in the occurrence of the PL phenomenon. PL spectroscopy is a highly precise method for evaluating the photoelectric characteristics of semiconductor materials. It is a non-invasive testing technique that does not damage the sample, making it a valuable tool in both the semiconductor industry and research domains. The fluorescence spectrum of light exhibits varying wavelengths across different semiconductor materials, thereby serving as a direct indicator of the sample’s structure and composition. Furthermore, as previously stated, the energy band structure and forbidden band width of ternary semiconductor alloy materials vary with changes in their components. Therefore, the proportion of components can be identified by examining the forbidden band width through PL testing. The PL spectra of various monolayer 2D alloy materials are presented in Figure 12. The spectra demonstrate the variation of component content and reveal that continuous adjustment can be achieved within the ranges of 670–800 nm, 620–680 nm, and 760–780 nm. That is, in the content variation range of MoS_2(1−x)_Se_2x_ from x = 0 to x = 1, the PL peak position underwent a continuous red-shift phenomenon, and the band gap opening became smaller and smaller. In contrast, the PL peaks of Mo_1−x_W_x_S_2_ and Mo_1−x_W_x_Se_2_ continued to blue-shift as the content of the components became larger, indicating that the forbidden band widths became larger as the component x increased. In summary, the application of PL spectroscopy can be utilised to acquire data on the forbidden band width of a sample. This information can be further examined in the area of regulated band gap engineering. In addition to PL spectroscopy, stoichiometry can be determined by using XRD, energy-dispersive X-ray spectroscopy (EDS), electron energy loss spectroscopy (EELS), XPS, chemical analysis, and other methods.

## 3. Photovoltaic Properties and Applications of 2D TMDs Alloy Materials

The precise regulation of energy bands in 2D semiconductor materials is necessary to fulfil the requirements of multifunctional materials in the optoelectronics field. Alloying engineering offers the advantage of continuously adjusting band edges, band gaps, and lattice constants, making it a popular area of interest among researchers. Transition metal materials within the same subgroup tend to form alloys and other compounds due to their similar lattice symmetry and low degree of lattice mismatch. As a result, a variety of transition metal sulphide compound alloy materials, such as Mo_1−x_W_x_S_2_ and Mo_1−x_W_x_Se_2_, have been extensively studied and reported. This section provides a comprehensive analysis of the development of 2D TMDs alloy materials. It covers a range of topics, including optoelectronic properties and their applications in hydrogen evolution reaction catalysis, field-effect transistors, lithium-sulphur battery catalysts, lasers, and other fields. The discussion is presented in a systematic manner.

### 3.1. Optoelectronic Properties of 2D TMDs Alloy Materials

**Figure 13:**
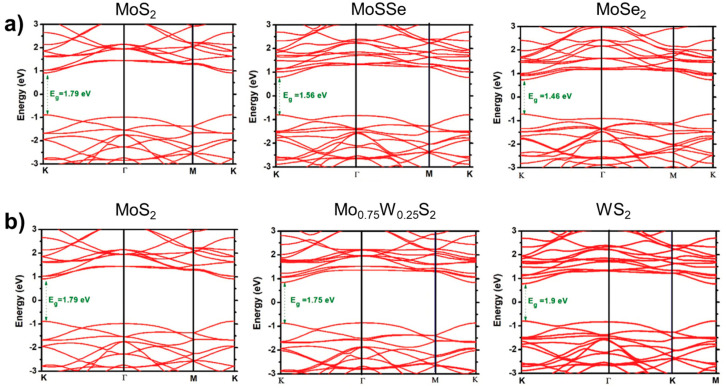
Band structures are shown for the TMD-ML alloys. (**a**) MoS_2(1−x)_Se_2x_ with x = 0.0, 0.5, and 1.0 and (**b**) Mo_1−x_W_x_S_2_ with x = 0.0, 0.25, and 1.0. Reproduced under the terms of the CC-BY 4.0 license [55]. Copyright 2021, The Author(s). Published by IOP Publishing Ltd. on behalf of the Institute of Physics and Deutsche Physikalische Gesellschaft.

The single-layer 2H phase MoS_2_ and WS_2_ are direct band gap semiconductor materials that emit light at approximately 650 nm and 590 nm bands, respectively. The single-layer MoS_2_ is suitable for direct application in field-effect transistors. The structure and properties of WS_2_ and MoS_2_ are similar, enabling them to form an alloy phase easily. However, current scientific research on Mo_1−x_W_x_S_2_ is limited. The present scientific research on Mo_1−x_W_x_S_2_ primarily concentrates on the development and adjustable band gap efficiency of single-layer or few-layer alloys. In terms of alloy growth, since the earliest 2D alloy is generally obtained by mechanical stripping [52,53,54], monolayer samples of 2D alloy materials were also obtained by mechanical stripping [16], but the stripped samples were too small in size and wasted, after which researchers obtained monolayer samples by using CVD, CVT, and ALD to prepare large-area, high-quality Mo_1−x_W_x_S_2_ 2D alloys with gradient layer structures [18,23]. Figure 13 shows the band structures of MoS_2(1−x)_Se_2x_ and Mo_1−x_W_x_S_2_ [55].

During the characterisation process of the 2D alloy material, the AFM was used to determine that the thickness of the monolayer Mo_1−x_W_x_S_2_ alloy material was approximately 0.7 nm. Additionally, the SEM detected a consistent distribution of elements on the surface of the monolayer alloy material. The SEM also observed a uniform distribution of elements on the surface of the monolayer alloy material; Raman spectroscopy showed the presence of two characteristic modes, MoS_2_-like E′ and WS_2_-like E′, as well as the A′ mode, which was blue-shifted with increasing tungsten (W) fraction because the A′ mode is only related to the vibrations of sulphur (S) atoms, while the E’ mode exhibits a bimodal behaviour attributed to the combined vibrations of the metal and S atoms [36,56]. The atomic structure (ordered, disordered, or clustered) of the material was observed using scanning transmission electron microscopy (STEM), and it was found to have a disordered distribution [57]. The PL spectra exhibited two distinct peaks, namely the A and B peaks. These peaks demonstrated a red-shift followed by a blue-shift in energy as the W component increased. The energy range of these peaks varied between 1.8 eV–2 eV and 2 eV–2.4 eV. Additionally, the energy band bending factors for the A and B peaks were 0.25 eV and 0.19 eV, respectively, as reported in [16]. In contrast, for the laminar gradient alloys, the absorption spectra showed a wide absorption range of 1.2 eV–2.5 eV. Laminar gradient alloys exhibited favourable optoelectronic characteristics, producing a photocurrent that is 3–4 times greater than that of unalloyed 2D materials [23]. Stability analysis of the monolayer Mo_1−x_W_x_S_2_ alloy material was performed theoretically using density flooding theory [58], which yielded negative formation energies and thus determined that the Mo_1−x_W_x_S_2_ alloy is stable under general conditions. Also, they predicted the existence of two ordered phases (x = 1/3, x = 2/3) in the monolayer Mo_1−x_W_x_S_2_ alloy.

### 3.2. Phase Changes

**Figure 14:**
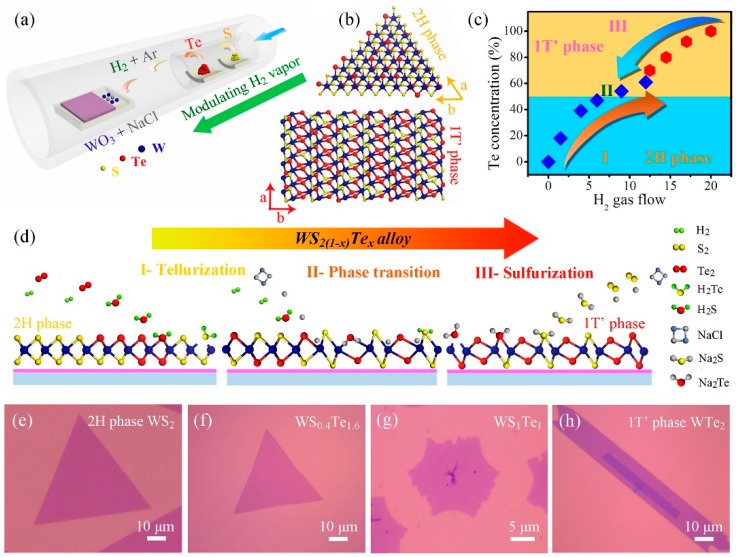
(**a**) Schematic illustration of the WS_2(1−x)_Te_2x_ CVD system. (**b**) Crystal structures of 2H and 1T′ phase WS_2(1−x)_Te_2x_ alloys, showing the distinguishing atomic configurations. (**c**) Summary of the Te concentration and the phase evolution as a function of the amount of H_2_ gas flow. (**d**) Growth schematic for preparing 2H and 1T′ phase WS_2(1−x)_Te_2x_ alloys in our work. Part I and III: the evolutions of the telluriding (or sulphuring) process by increasing (or decreasing) the H_2_ environment. Part II: the phase conversion from 2H to 1T′ phase with the increase in H_2_ gas. (**e**–**h**) OM images of 2H phase WS_2_, WS_0.4_Te_1.6_, 1T′ phase WS_1_Te_1_ and WTe_2_ domains, respectively. Reproduced with permission [60]. Copyright 2019, Elsevier.

In addition to stable phases, TMDs have a number of substable phases with unique optical and electrical properties. By realising controlled phase transitions between different crystalline phases, TMDs exhibit some superior properties and new potentials in electronic and optoelectronic applications [59]. Wang et al. reported a CVD synthesis method. They synthesised monolayer WS_2(1−x)_Te_2x_ alloys with different Te ratios by modulating H_2_ gas. A structural phase transition from the semiconducting 2H phase to the semi-metallic 1T′ phase at high Te ratios was achieved, and the optical band gap was red-shifted from 1.97 eV to 1.67 eV. The conductive behaviour of the device was effectively modulated (Figure 14) [60]. Zhang et al. achieved a 2H to 1T phase transition of MoS_2x_Se_2(1−x)_ and Mo_x_W_1−x_S_2_ through Li-ion intercalation [61]. In addition, Amey Apte et al. achieved the 2H to 1T phase transition by applying stress to a MoWSe_2_ alloy and verified the process using molecular dynamics simulations and in situ electron beam exposure with high-angle annular dark-field (HAADF) STEM imaging [62].

Phase transitions achieved by using electric field guidance were reported by Zhang et al. Structural transitions from 2H semiconductors to distorted transient structures (2Hd) and rhombohedral crystal Td conducting phases were achieved for resistive random-access memory (RRAM) devices based on 2H-MoTe_2_ and Mo_1−x_W_x_Te_2_. It was shown that controlled electrical state switching in 2D materials is achievable [63].

### 3.3. Application of 2D TMDs Alloy Materials

Alloyed 2D materials possess adjustable properties and optoelectronic characteristics that surpass or are absent in the original 2D materials, as indicated in the above review. This subsection provides a brief introduction to the application of hydrogen precipitation catalysis and field-effect transistor devices.

#### 3.3.1. Hydrogen Evolution Reaction (HER)

The production of clean energy (hydrogen) relies heavily on the development of stable and efficient HER catalysts. TMDs have gained significant attention in the field of photoelectrochemical (PEC) water decomposition due to their unique electronic and optical properties. These materials hold promise for efficient and sustainable hydrogen production through sunlight-driven water splitting, which is a crucial step in renewable energy generation and storage [64,65,66,67,68]. The HER is a crucial half-reaction in electrochemical and photoelectrochemical water-splitting processes. The electroactivity of TMDs can be modulated by changing their electronic structure, and the HER activity is dependent on the proportion of the alloy.

**Figure 15:**
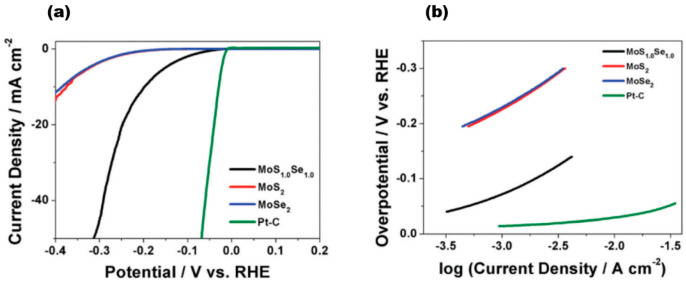
(**a**) IR-corrected linear sweep voltammograms of MoS_2(1−x)_Se_2x_; (**b**) Tafel plots corresponding to MoS_2(1−x)_Se_2x_ and Pt–C. Reproduced with permission [15]. Copyright 2014, Royal Society of Chemistry.

Zhu et al. achieved excellent catalytic activity, long-term stability, and universality based on MoS_2_ under both acidic and alkaline conditions [69]. 1T′-WTe_2_ nanoribbons have also been reported to exhibit ultrahigh stability over 5000 cycles and 20 h at 10 mA/cm^2^ current [70]. The long-term stability of TMD alloy materials has also been demonstrated. The VSSe sample reported by Hu et al. exhibited a lower overpotential of −180 mV at a current density of 10 mA cm^−2^, a Tafel slope of 87 mV dec^−1^, and better durability [71]. Furthermore, the conductivity of the catalyst material is a critical factor in determining its catalytic performance. Among the reported MoS_2(1−x)_Se_2x_ [15] alloy materials, Figure 15a represents a linear scanning voltammogram of MoS_2(1−x)_Se_2x_, where the red line represents MoS_2_ (x = 0), the blue line represents MoSe_2_ (x = 1), the black line represents MoS_1.0_Se_1.0_ (x = 0.5), and the green colour represents Pt-C. The electrochemical activity exhibits diversity as the components vary. The data clearly show the positive effect of Se in the MoS_2_ lattice or S in the MoSe_2_ lattice on HER. Upon analysing the HER onset potential shift of few-layer MoS_1.0_Se_1.0_ in comparison to the few-layer MoS_2_ and MoSe_2_ nanosheets, it was observed that the alloyed material consistently outperforms the pure sulphide and selenide variants. Notably, MoS_1.0_Se_1.0_ exhibited the highest efficacy among all combinations. Moreover, the Tafel slope, a parameter used to evaluate the HER electroactivity and mechanism, was determined from the linear region of the data shown in Figure 15b, and it can be seen that the Tafel slope is 96 mV dec^−1^ for MoSe_2_, 95 mV dec^−1^ for MoSe_2_, and about 56 mV dec^−1^ for MoS_1.0_Se_1.0_. For a constant load of 180 μg cm^−2^, the exchange currents obtained from the polarisation curves were 320 μA cm^−2^ for MoS_1.0_Se_1.0_, 45 μA cm^−2^ for MoSe_2_, and 36 μA cm^−2^ for MoS_2_, indicating the highest HER activity of MoS_1.0_Se_1.0_ compared to MoSe_2_ and MoS_2_. The collective findings indicate that effective doping of materials results in a significant increase in their HER activity. Additionally, modifying the composition of layered sulphide generics to fine-tune their electrochemical activity positively impacts the photocatalytic hydrogen precipitation on 2D materials.

Kang et al. synthesised alloy nanosheets of Re_1−x_Mo_x_Se_2_ (x ranging from 0 to 100%) by using a hydrothermal reaction. A phase transition evolution process of 1T″->1T′->2H occurred as x increases. Their results showed that the HER catalytic activity depends on x, with the best catalytic activity at x = 10% [72]. They synthesised (MoWV)Se_2_ nanosheets with different compositions by using another colloidal reaction in 2023, with the molar fraction of V atoms (X_V_) increased to 0.8, which succeeded in generating a highly hydration-resistant metallic phase. And the phase transition from 2H to 1T occurred when the X_V_ value was in the range of 0.62–0.75. They demonstrated that the HER properties improve with the increase in X_V_ [73].

#### 3.3.2. Field-Effect Transistor (FET)

**Figure 16:**
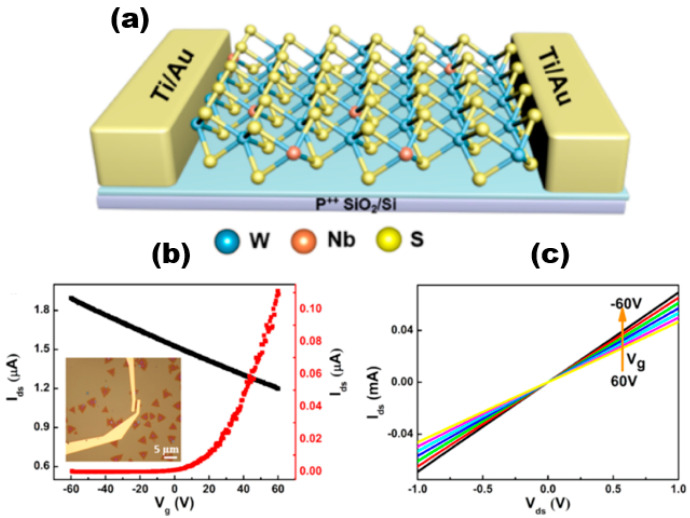
(**a**) Schematic of FET based on monolayer Nb_x_W_1−x_S_2_; (**b**) I_ds_ and V_g_ characteristics of single-layer WS_2_ and Nb_x_W_1−x_S_2_ FET devices at different V_ds_. (**c**) Current output curves of single-layer Nb_x_W_1−x_S_2_ at different voltage values. Reproduced with permission [74]. Copyright 2019, American Chemical Society.

Alloyed 2D TMDs materials exhibit more unique electrical properties than 2D TMDs. Liu et al. [74] successfully synthesised Nb_x_W_1−x_S_2_ alloy materials and constructed field-effect transistor devices based on the alloy materials. The electrical measurements conducted indicated that the addition of Nb atoms to WS_2_ resulted in the manifestation of P-type semiconductor characteristics. The incorporation of Nb dopants could effectively modulate the band gap of Nb_x_W_1−x_S_2_ and enhance the optical properties of WS_2_. The carrier type of WS_2_ underwent a transition from intrinsic n-type to p-type upon Nb doping, which was a significant observation. Figure 16a shows the schematic diagram of the Nb_x_W_1−x_S_2_-based FET device. The electrical transport characteristics of WS_2_ and Nb_x_W_1−x_S_2_ FETs were obtained by using electron beam lithography and vapour deposition of the sample with Ti/Au (10/30 nm) contacts and measured, and the inset shows the optical image of the Nb_x_W_1−x_S_2_ FET device. The non-linear output of monolayer WS_2_ was dependent on the source drain current (I_ds_) and the n-type gate voltage (V_g_), indicating the existence of a Schottky barrier between the Ti-WS_2_ contacts. And the Ids showed a decreasing trend with increasing V_g_ after Nb doping, which indicated that Nb_x_W_1−x_S_2_ is heavily p-type-doped. The Ids stayed at a high value and did not saturate when V_g_ was adjusted, indicating a better Nb doping effect. And this phenomenon can be explained by the tunnelling transport mechanism [75,76]. The rise in conductive current observed in Nb_x_W_1−x_S_2_ can be attributed to the increase in hole concentration resulting from the doping process. This increase in hole concentration leads to a linear increase in Ids with V_ds_, as illustrated in Figure 16c, when Nb is used as the dopant.

#### 3.3.3. Lithium-Sulphur Battery Catalysts

**Figure 17:**
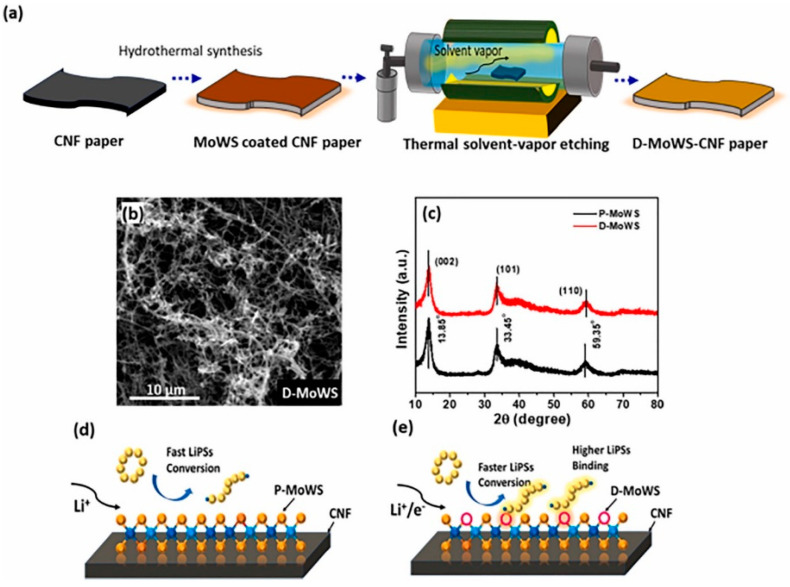
(**a**) Schematics illustrating the synthesis process of hydrothermally grown MoWS catalyst on CNF paper and its defect engineering process. Low-magnification FESEM image of (**b**) defect-engineered (D-MoWS) catalysts on CNF paper. (**c**) XRD analysis of P- and D-MoWS catalyst on CNF. (**d**,**e**) A schematic of P- and D-MoWS-CNF structure representing the effect of defect engineering on polysulphide binding and conversion. Reproduced with permission [11]. Copyright 2021, Elsevier.

As one of the most promising next-generation energy storage systems, lithium-sulphur batteries have the advantages of high theoretical energy density, higher energy density, abundant resources, and high safety [77,78]. However, their practical applications are limited by the growth of lithium dendrites and lithium polysulphide shuttles. In order to solve LiPS-related conversion problems, catalyst materials such as transition metal oxides, sulphides, nitrides, carbides, etc., are needed. TMDs, as relatively stable catalyst materials, can avoid side reactions in the battery cycle and improve the conversion of LiPSs. Bhoyate et al. in 2020 showed that LiPSs can be converted to LiPSs through a two-step process of co-sputtering and sulphurisation to synthesise a 2D Mo_0.5_W_0.5_S_2_ alloy with 2H (semiconducting)-1T (metallic) mixed phase and confirmed the higher LiPS binding effect and catalytic performance by using electrochemical analysis [11]. They further synthesised the mixed 2H + 1T phase 2D MoWS alloy catalysts in 2021 by using the method of hydrothermal synthesis and defects engineering, which resulted in a high charge transfer and LiPSs conversion. The D-MoWS catalysts deposited on CNF paper maintained structural homogeneity during the etching process (Figure 17). The LSB anode with high sulphur loading (10 mg cm^−2^) presented a high area capacity of 7.6 mAh cm^−2^, high cycling stability (100 cycles), and a low N/P ratio (1.7) at 0.3C. By comparing the battery performance (within 10 cycles) of the bare CNF-S cathode with the same sulphur loading of 10 cm^−2^, it was demonstrated that the catalyst efficiently utilises sulphur and reduces the N/P ratio, significantly increasing the weight energy density up to the highest reported value of 1090 Wh kg^−1^ [11]. Feng et al. used MoWS_2_@MXene@CNT composite material as the main cathode material for L-S batteries, in which MoWS_2_ acted as a polar substance to accelerate polysulphide conversion, MXene enhanced the electron conductivity, and CNT accelerated the electron transfer rate. The composite material showed good multiplicity and cycling stability as an electrode material [12].

#### 3.3.4. Lasers

**Figure 18:**
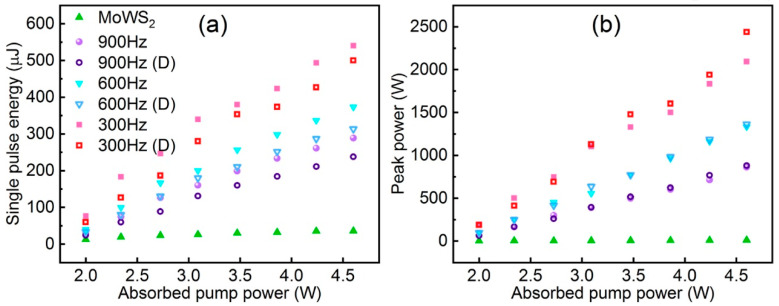
(**a**) Single pulse energy and (**b**) pulse peak power of Q-switching envelope versus absorbed pump power. Reproduced with permission [83] Copyright 2022, Elsevier.

The application of TMDs as saturable absorbers (SAs) for Q-switched operation in fibre lasers has been explored and investigated due to their strong photoluminescence, ultrafast carrier dynamics in monolayer and few-layer forms, and semiconducting ability to have a tuneable band gap from the visible to the near-infrared region [79,80]. Alloying TMD materials by adjusting the composition of metallic (MX2) or sulphur (X) elements allows for more precise tuning of the energy band structure of TMDs to better exploit their potential as optoelectronic devices. TMD alloys likewise offer a variety of advantages that are highly favourable for saturable absorption applications, as well as good thermodynamic and environmental stability. Wang et al. in 2018 demonstrated the use of Mo_0.5_W_0.5_S_2_ as an optoelectronic device by using a microwave-assisted solvothermal method to fabricate Mo_0.5_W_0.5_S_2_ and reported for the first time the use of Mo_0.5_W_0.5_S_2_ polymer film and tapered fibre as SA for Q-switched Yb-doped fibre lasers. The modulation depth and saturable intensity of the film SA were 5.63% and 6.82 MW cm^−2^. A Mo_0.5_W_0.5_S_2_-PVA film SA yielding Q-switched pulses with a pulse energy of 148.8 nJ was realised. The minimum pulse duration was 1.22 μs. A higher pulse energy of 339 nJ and pulse width of 1.46 μs and a higher pulse energy of 339 nJ and pulse width of 1.46 μs were also obtained with the fibre-taper Mo_0.5_W_0.5_S_2_ SA. In addition, they compared Mo_0.5_W_0.5_S_2_ SA with MoS_2_ and WS_2_ SA, and Mo_0.5_W_0.5_S_2_ SA had a narrower pulse width and was superior in terms of single-pulse energy [13]. Ahmad et al. were able to generate stable Q-switched pulses in the 1.0 μm, 1.5 μm, and 2 μm regions using MoWSe_2_ alloy as an all-fibre passively Q-switched Yb-, Er-, and Tm-doped fibre laser. MoWSe_2_ SA performance did not show any degradation over time, demonstrating its potential and applicability as a novel 2D broadband SA material [81]. Yin et al. reported the first realisation of femtosecond photonics of Mo_0.5_W_0.5_S_2_ SA in 2022, suggesting that it is a good candidate for the mode-locked ultrafast fibre laser for optoelectronic and optical communication applications [82]. Niu et al. prepared 2D MoWS_2_ by using the CVD method and liquid-phase glass method and proposed an LD-pumped doubly Q-switched mode-locked (QML) Tm:YAP laser at a waveband using MoWS_2_ SA and EOM. In comparison to the single QML laser with MoWS_2_ SA or EOM, the laser could produce a shorter pulse duration and higher peak power (Figure 18) [83].

#### 3.3.5. Others

Due to their excellent properties, 2D alloyed TMDs have many promising applications in other fields. Ghazanfari et al. prepared for the first time highly sensitive and selective electrochemical sensors based on screen-printed electrodes (MoWS_2_/SPE) modified with MoWS_2_ nanoparticles. The MoWS_2_/SPE showed excellent performance in both electrocatalytic activity and selectivity determination and has been demonstrated to be suitable for the determination of target analytes in tap water and food samples [84]. Ko et al. analysed the effect of the Mo_x_W_1−x_S_2_ microstructure on resistance and the temperature coefficient of resistance (TCR) by using I–V measurements. They vulcanised MoW alloys at 800–850 °C to convert the metal layers into Mo_x_W_1−x_S_2_ compound semiconductor films. They measured the temperature-dependent I–V characteristics of the samples in the range of 290–570 K and calculated the resistance based on the results. The well-crystallised Mo_x_W_1−x_S_2_ thin films after sulphurisation at 950 °C showed a TCR as high as –1.633%/K, which demonstrated their potential for further development and application in thermal sensors [85]. The realisation of superlubrication at metal–metal friction interfaces is a bottleneck that needs to be solved in modern industry. Jiang et al. reduced the coefficient of friction of steel–steel in dry argon to 0.008 by using a graphene and MoWS_4_ nanosheets heterojunction, which reduces metal-to-metal adhesion and direct contact and provides a new research route for solid lubricant development. In addition to the traditional TMD film structure, there are also 2D molecular layer vertically assembled TMDs [86]. Compared to the film structure, vertically assembled TMDs better expose the layer edge position rather than the basal surface and show anisotropic carrier transport properties. This vertically assembled form provides a feasible idea for the development and application of a TMD alloy. Heterojunctions in which the band gap of one semiconductor lies entirely within the band gap of another semiconductor are type I heterojunctions, which are preferred for luminescence applications. Zhou et al. demonstrated that type I heterojunctions can be realised through the use of interlayer alloying and/or monolayer rotation by means of an ab initio density functional theory approach [87]. He et al. provided high reversible capacities of 517.4 and 362.4 mAh g^−1^ at 100 and 1000 mA g^−1^, respectively, when optimised vacancy-rich MoSSe alloys were used as anodes for potassium ion batteries. [88]

In accordance with the above applications, TMD alloys are yet to be developed for applications in imaging [89], metamaterials [90], etc., which are areas also in need of development.

## 4. Conclusions

Two-dimensional TMDs materials have attracted much attention in the fields of microelectronics, catalysis, and optoelectronic devices because of their tuneable band width, carrier migration type, excellent electrical and thermal conductivity, and rich optoelectronic properties. We present a comprehensive review of 2D TMDs alloy materials, including their development and characterisation methods, optoelectronic properties, and applications. In addition to HER and FET, their potential for development in areas such as batteries, lasers, imaging, lubricants, etc., cannot be underestimated. Opportunities and obstacles for future study in the field are focused on the following:

- The research on 2D alloy materials, such as transition metal-sulphur compounds, focuses on the formation of single-layer or few-layer alloy materials, the regulation of band gap and carrier migration types, electrochemical hydrogen precipitation, and optoelectronic devices. Research on bulk phase alloy materials is relatively scarce, and there is an urgent need to find new outlets for applications.
- Insufficient research has been conducted to investigate the preparation of 2D alloy materials with special morphology or morphology control, as well as their corresponding optoelectronic properties.
- The existing approach for regulating alloy concentration involves a singular method that is determined solely by the feeding ratio. In the field of catalysis, it is necessary to explore methods of alloy doping that can increase the number of active sites. Additionally, it is recommended to develop single-atom catalysts.
- Maintaining the enduring stability and dependability of TMDs alloys for diverse applications like electronics and catalysis continues to pose a challenge, particularly in demanding conditions.

TMDs alloy materials have seen compelling progress. They have a wealth of development opportunities in applications such as flexible electronics, quantum technology, heterostructures, and biomedical sensing. However, challenges remain in terms of precise compositional analysis, defect characterisation, scalability, stability, and integration, which must be overcome to realise their full potential in various fields.
